# Are Anti-Retinal Autoantibodies a Cause or a Consequence of Retinal Degeneration in Autoimmune Retinopathies?

**DOI:** 10.3389/fimmu.2018.00765

**Published:** 2018-04-16

**Authors:** Grazyna Adamus

**Affiliations:** School of Medicine, Casey Eye Institute, Oregon Health & Science University, Portland, OR, United States

**Keywords:** autoantibody, retinal degeneration, cancer-associated retinopathy, melanoma-associated retinopathy, recoverin, enolase, transient receptor potential channel protein 1, epitope mapping

## Abstract

Autoantibodies (AAbs) against various retinal proteins have been associated with vision loss in paraneoplastic and non-paraneoplastic autoimmune retinopathies (AR). There are two major paraneoplastic syndromes associated anti-retinal AAbs, cancer-associated retinopathy (CAR), and melanoma-associated retinopathy. Some people without a cancer diagnosis may present symptoms of CAR and have anti-retinal AAbs. The etiology and pathogenesis of those entities are not fully understood. In this review, we provide evidence for the role of AAbs in retinal death and degeneration. Studies of epitope mapping for anti-recoverin, anti-enolase, and anti-carbonic anhydrase II revealed that although patients’ AAbs may recognize the same retinal protein as normal individuals they bind to different molecular domains, which allows distinguishing between normal and diseased AAbs. Given the great diversity of anti-retinal AAbs, it is likely some antibodies have greater pathogenic potential than others. Pathogenic, but not normal antibodies penetrate the target cell, reach their specific antigen, induce apoptosis, and impact retinal pathophysiology. Photoreceptors, dying by apoptosis, induced by other than immunologic mechanisms produce substantial amounts of metabolic debris, which consequently leads to autoimmunization and enhanced permeability of the blood–retinal barrier. AAbs that were made as a part of anti-cancer response are likely to be the cause of retinal degeneration, whereas others, generated against released antigens from damaged retina, contribute to the progression of retinopathy. Altogether, AAbs may trigger retinal degeneration and may also exacerbate the degenerative process in response to the release of sequestered antigens and influence disease progression.

## Introduction

**Figure 1:**
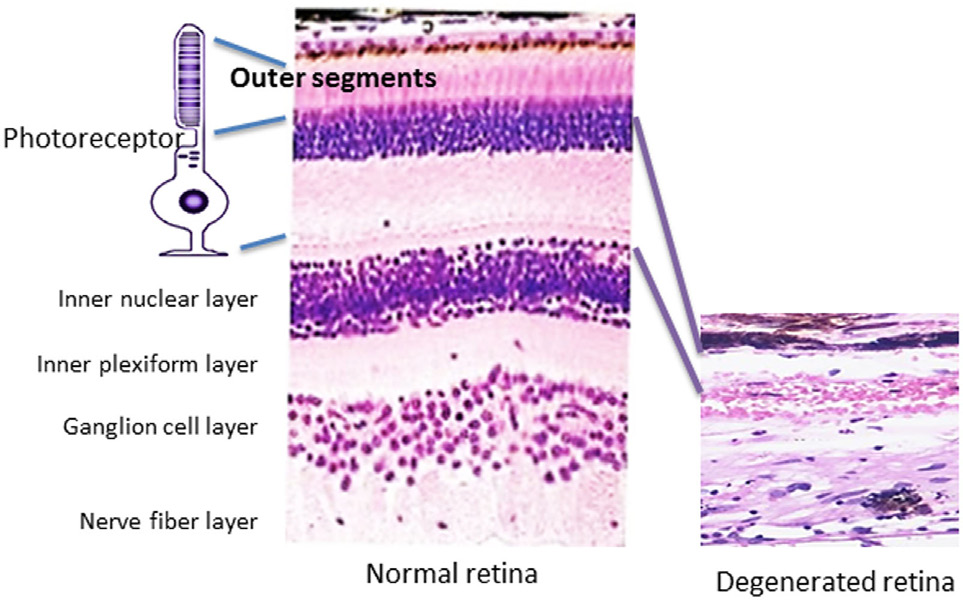
Pathology of retinal degeneration. A picture that illustrates a rod photoreceptor cell next to normal human retina (on the left) that is composed of several cell layers as follows: inner and outer segments of rod and cones of photoreceptors cells, outer nuclear layer, outer plexiform layer, inner nuclear layer, inner plexiform layer, ganglion cells layer, nerve fiber layer (axons of ganglion cells), and outer limiting membrane that neighbors with vitreous. A photograph of degenerated retina from a patient who lost completely photoreceptor cell layer (on the right), only inner part of the retina remained unaffected.

Autoantibodies (AAbs) against retinal proteins have been associated with vision disturbance in paraneoplastic and non-paraneoplastic autoimmune retinopathies (AR). Cancer-associated retinopathy (CAR), a visual paraneoplastic syndrome, is characterized by sudden and unexplained loss of vision associated with distant cancer and the presence of AAbs (1, 2). Those AAbs bind to various antigens present in the retina and are collectively called anti-retinal AAbs. CAR can be associated with any cancer and dysfunction of photoreceptor cells and other retinal cells (3). A disorder that affects people with metastatic skin melanoma and vision loss related to the dysfunction of retinal bipolar cells has been separated from CAR syndrome and was named melanoma-associated retinopathy (MAR) (4–6). Autoimmune retinopathy (AR) concerns people with acute or subacute vision loss who present symptoms of CAR and have anti-retinal AAbs but without a cancer diagnosis at presentation (7). Autoimmune retinopathies commonly affect individuals of over 50 years old. As the general population grows older, the number of seropositive patients for anti-retinal AAbs increases with complaints of vision loss. In this review, we will provide evidence for autoimmunity as a primary contributing mechanism underlying retinal cell death or as a secondary role in exacerbating the degenerative processes in response to antigens being released from the degenerating retina, thus influencing the progression of AR. Even if AAbs did not initiate the pathogenic processes, they could still drastically impact the progression of AR. Pathology of retinal degeneration in human tissue is illustrated in Figure 1.

## Autoimmune Retinal Degeneration Syndromes

Paraneoplastic (CAR, MAR) and non-paraneoplastic (AR) disorders are immunologically and symptomatically heterogeneous. CAR is characterized by sudden and progressive loss of vision associated with photosensitivity, reduced visual acuity, defects in color vision, constriction of visual fields, ring scotoma, and attenuated retinal arteriole (1, 2). The function of both cone and rod photoreceptor cells can be affected. In the case of cone-related dysfunction, patients present with photosensitivity, photopsias, glare, severely reduced central vision, and impaired color perception. Rod dysfunction manifests with night blindness, impaired dark adaptation, and peripheral visual field loss, e.g., ring scotoma.

Melanoma-associated retinopathy is characterized by symptoms of acquired night blindness, light sensations, visual loss, defect in visual fields, and reduced b-waves in the electroretinogram (ERG) (8). It primarily affects bipolar cell function, but photoreceptors can also be damaged in MAR patients (9, 10). Despite significant variations in signs and symptoms, we and others have found some similarities in the clinical presentation of retinopathy associated with AAbs of the same specificity. For example, the clinical phenotype for anti-recoverin-associated retinopathy appears to be different from the retinopathy that is associated with anti-enolase antibodies (11–13). These disorders are rare. The low incidence of CAR and MAR could be related to the fact that most patients with carcinomas or melanoma do not perceive that their eye problems could be connected to their distant malignancy. It may also be possible that substantial subjective vision loss only occurs after a critical number of rod and cone photoreceptors are destroyed (14–16). Retinal autoimmunity can also contribute to photoreceptor cell loss in hereditary retinitis pigmentosa (RP) and possibly other inherited retinal dystrophies.

## Generation of Anti-Retinal AAbs

**Figure 2:**
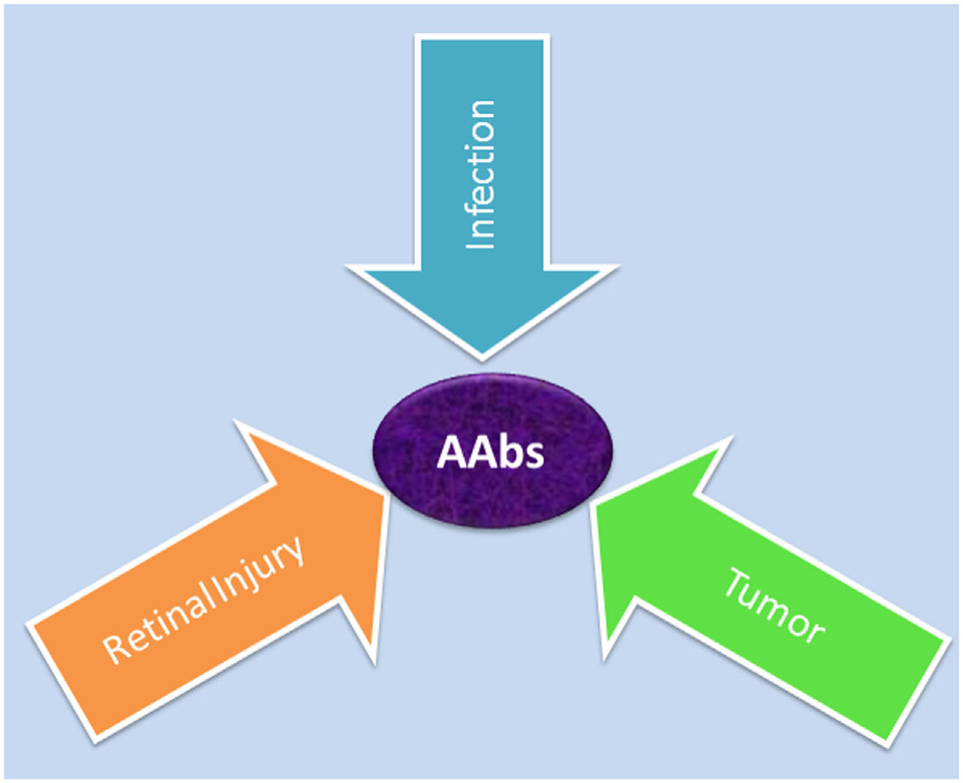
Possible causes for generation of autoantibodies reacted with retinal antigens: anti-microbial responses (infection) against similar antigens released after infection, antitumor responses (tumor) against upregulated similar proteins, or anti-retinal responses to released sequestered proteins (retinal injury) from dying retinal cells.

Autoantibodies against a variety of retinal antigens have been detected in patients’ blood but the precise mechanism for AAb origination remains unclear. Specifically, the question is whether the antibodies are involved in the initial pathogenesis of retinal disease or if they develop during the course of the disease as a secondary event. We contemplate three possible causes of AAb formation in retinal degenerative diseases: (A) the antitumor response, (B) the anti-microbial response, or (C) the autoimmune response against self-antigens that are released from a damaged retina (Figure 2).

(A) There is evidence that CAR antibodies are produced as a part of antitumor response (17–22). Both malignant and benign tumors are capable of inducing a humoral immune response (23, 24). During antitumor response, released antigens are picked up and processed by antigen-presenting cells, which eventually leads to the production of AAbs that may cross-react with protein antigens in the retina. Some retinal antigens are identical or partially identical to the antigens present in cancer cells that are upregulated during tumorigenesis. An example of such a response are AAbs against recoverin, a protein normally sequestered in the retina but it is also produced in small cell carcinoma of the lung and other cancers (25–29). The process of AAb formation likely starts in the pre-malignant phase and may serve to limit the tumor growth (30, 31). Anti-recoverin AAbs were also found in patients that initially presented with ocular disturbances—without cancer; however, a malignancy was later discovered (25, 32, 33).
(B) The second possibility for AAb generation is an anti-microbial response, since there are putative similarities between proteins that exist in pathogens and the retina. An autoimmune response can emerge following infection by a microbial pathogen, whose proteins have structural similarities to human proteins (34). Proteins, to which the immune system is normally self-tolerant, might elicit autoimmune responses. Thus, antibodies evoked against pathogenic proteins can cross-react with retinal proteins and act as AAbs and the implicated autoantigens then provide a source for persistent stimulation. Exposure to infections by bacteria and viruses throughout the entire life of the host induces memory microbe-specific T and B cells, which then recognize self-antigens of the eye, leading to autoimmune processes. Cross-reactive microbial AAbs with retinal antigens, in particular, against conserved proteins with important physiological functions can cross the blood–retinal barrier (BRB) and access similar or identical antigens in retinal cells (35–37). An example of such cross-reactive antibodies is AAb against glycolytic enzymes, which have important metabolic functions in both microbial and retinal cells. Moreover, glycolytic proteins are multifunctional, they not only exist in the cytosol, but are also exposed on the membrane of microbes, making them easily accessible to the immune system (36).
(C) The third mechanism for antibody production can be explained by the availability of retinal antigens during retinal degeneration initiated by some other the immunological processes. There is evidence for the activation of autoimmune responses secondary to retinal degeneration, due to the genetic mutation in different forms of RP (38–40). Causative mutations may initiate cellular stress in photoreceptor cells through the secretion of chemokines and the recruitment of microglia into the outer retina (41). It has been hypothesized that retinal proteins that are sequestered under normal physiological conditions, and protected from immune recognition, are released from the damaged tissue and become targets of the immune effector functions once exposed to the immune system (42, 43). The concept that new AAbs are produced in response to debris from regions of pathology implies that these AAbs function in the clearance of debris from the tissue (44). A variety of AAbs have been detected in patients with retinal diseases, but not all of them are against proteins that are normally present in photoreceptor cells, especially in outer segments. The potential antigenic proteins participate in the phototransduction process, by which the photoreceptor cells generate electrical signals in response to the absorption of photons (45). Photoreceptor cells are post-mitotic, terminally differentiated retinal cells that have no regenerative ability under normal physiological conditions, whose number inversely decreases with age—presumably due to cell death (46). Retinal cell death is also associated with increased numbers of subretinal microglial accumulation and complement activation (47). Although the eye has an immune privileged status, it is still susceptible to immune-mediated inflammatory disease, both by infectious and autoimmune stimuli (48, 49). A crucial step in the activation process is the recruitment of inflammatory cells, such as macrophages and microglia, to the local injured area/retina; which leads to the release of pro-inflammatory cytokines and amplifies the disease’s process (50). The accumulation of debris in the outer segment provides a signaling mechanism for the activation and chemotaxis of microglial cells (41). Such processes may enhance disease progression by augmenting apoptotic photoreceptor cell death, disrupting the BRB, and attracting blood macrophages into the retina (51, 52). Excessive deposition of complement components at the retinal pigment epithelial/outer segments of photoreceptor cell space, particularly when concentrated in deposits of metabolic debris, may act as a trigger for inflammatory macrophage activation.

The research using the dystrophic Royal College of Surgeons (RCS) rat model showed that anti-retinal AAbs and T cells were generated, with distinctive activation trends, in response to the availability of antigenic material being released from dying photoreceptor cells during retinal degeneration (53). A strong initial response in anti-photoreceptor antibodies declined about the rat age 40 days, but later, there was a rebound, with a subsequent wave of antibody production, caused by an additional antigenic re-stimulation as more cells died and remained measurable until photoreceptor cells disappeared and self-antigens that were being released from dying cells were no longer available (53). This suggests that, even if AAbs did not initiate the pathogenic processes, they could still drastically influence their progression. Moreover, the adoptive transfer of anti-retinal AAbs obtained from RCS rats with inherited retinal degeneration induced disruption of the blood–retinal barrier, upregulation of MCP-1 (CCL2) chemokine, and attracted macrophages/microglia into the retina. This additional influx of microglia correlated with increased levels of photoreceptor apoptosis, thus influencing the long-term photoreceptor survival (53). It is likely that early on, the same anti-retinal AAbs could contribute to the cell death, and later, to the progression of retinal degeneration by recruiting activated macrophages/microglia into the retina and production of new AAbs.

Although AAbs are frequently found in the circulation of patients with loss of vision, it is difficult to determine the primary mechanism of antibody formation. Assessing the cross-reactivity between cancer-retina and microbial-retinal antigens, using tissue samples or purified antigens helps with identification of the source of antigenic stimulation. However, identifying AAbs that were generated as a result of retinal death is much more difficult task. Regardless of the origination of anti-retinal AAbs, they are found to persist over time in the circulation and are associated with a stable or progressive course of vision loss (11). Due to the chronic nature of retinal autoimmunity, AAbs are likely to appear before manifestation of clinical symptoms though it is not possible to test non-symptomatic patients. Such AAbs could provide a good predictive biomarker for the potential development of retinal disease and neoplasm.

## Retinal Autoantigens

**Figure 3:**
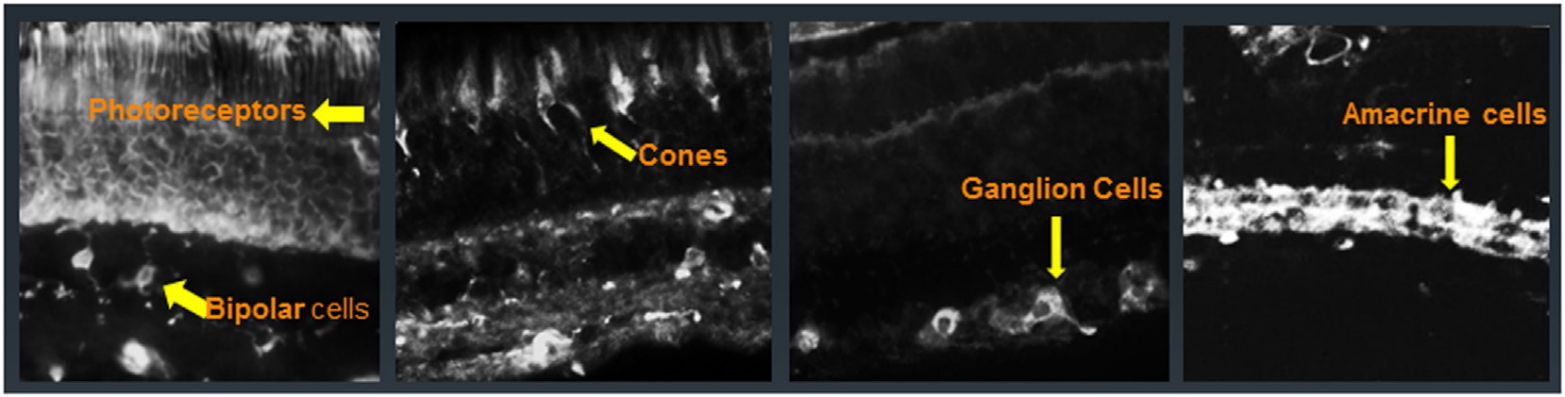
Immunofluorescent labeling of the human retina with cancer-associated retinopathy patients’ anti-retinal autoantibodies (AAbs) specific for different cellular structures. From left to right: AAbs label photoreceptor cells and bipolar cells, cone photoreceptors, ganglion cells, and amacrine cells; arrows point at immunofluorescent cells.

Patients with CAR, MAR, and AR have AAbs that generally target intracellular proteins, and only a few that are directed against membrane proteins, located in various retinal cell types (Figure 3) (54–58). Some of the first AAbs found in association with CAR are AAbs against recoverin, a calcium-binding protein that plays an important function in visual phototransduction (6, 59–61). Initially, only patients who had been diagnosed with small cell carcinoma of the lung were found to be seropositive for anti-recoverin AAbs (62, 63). However, in subsequent years, other malignancies were found to be associated with anti-recoverin AAbs (64). Anti-recoverin AAbs were also detected in patients with different cancers without visual presentation, but were not reported in healthy individuals without cancer, suggesting that AAbs are mainly generated against the cancer-expressed recoverin (65). Recoverin has been considered a main biomarker for CAR syndrome but anti-recoverin AAb presence is infrequent; in fact, only about 5% of CAR patients possess anti-recoverin antibodies (66). However, the absence of AAbs against recoverin does not exclude a diagnosis of paraneoplastic syndrome. Thus far, over 30 different antigens in the retina have been identified in association of vision loss (67–70).

Presumed targets in retinal degeneration are photoreceptor cells, the outer layer of the retina (Figure 1). Recoverin is a photoreceptor cell protein, in addition to several other photoreceptor antigens found, including arrestin, guanylate cyclase-activating protein, transducin-α and transducin-β, TULP1, Rab6, rhodopsin, and interphotoreceptor retinoid-binding protein (IRBP) (10, 22, 28, 54, 55, 66, 71, 72). However, sera of patients with CAR possess AAbs that not only react with photoreceptor cell antigens but also with bipolar and ganglion cells of the retina (62, 66, 73, 74). AAbs against glycolytic enzymes, such as enolase, aldolase, glyceraldehyde-3-phosphate dehydrogenase, and pyruvate kinase M2, dominate in sera of patients with AR (36). Antibodies against heat shock proteins, such as heat shock protein 27 (HSP27) and HSP65, have also been detected in patients with CAR and AR (66, 75, 76). Carbonic anhydrase II (CAII) is one of the major target proteins in prostate cancer-CAR and AR (77).

In MAR, transient receptor potential channel protein 1 (TRPM1), a membrane autoantigen is associated with retinal ON bipolar cell dysfunction (57, 58, 78–80). TRPM1 is also found in melanocytes (57, 58). The epitope of the TRPM1 AAbs is localized to the short intracellular domain in the amino terminal part of TRPM1 sequence (58, 80). It is hypothesized that TRPM1 AAbs are generated in response to abnormal TRPM1 polypeptides, encoded by an alternate mRNA splice variant that is expressed by malignant melanocytes (56). These AAbs are infrequent, found in less than 5% of MAR patients. More often, AAbs against proteins involved in phototransduction have been detected, likely because human melanoma cells *in vitro* express rhodopsin, transducin, and cyclic guanosine 3′,5′-monophosphate phosphodiesterase 6, guanylyl cyclase 1, recoverin, and arrestin (81). In fact, AAbs against transducin, rhodopsin, arrestin, and IRBP have been found in MAR in addition to α-enolase, CAII, myelin basic protein, mitofilin, and titin (4, 22, 28, 82, 83). In recent years, there have been multiple reports of a MAR-like retinopathy with associated detachments of the retinal pigment epithelium (RPE) and neurosensory retina. Such a clinical presentation is termed paraneoplastic vitelliform retinopathy. In some cases, three additional proteins, besides the usual anti-enolase-α, anti-bestrophin, and anti-peroxiredoxin, were detected in the serum (84–86).

Autoantibodies with similar specificities were found in patients with AR who do not have a previous history of visual problems and develop a sudden onset of photopsias, night blindness, scotomata, and visual field loss (39, 87). AAbs against recoverin, α-enolase, aldolase, and CAII as well as AAbs against heat shock proteins (HSP27, HSP60) and CRMP2 were identified in patients with AR (66, 76, 88, 89). It has been speculated that AR can also be a secondary complication of other conditions such as RP, birdshot retinopathy, or acute zonal occult outer retinopathy (AZOOR). RP patients with cystoid macular edema often have antibodies against CAII (90). High incidence of AAbs has been observed in patients with AZOOR but it is not clear whether this condition is autoimmune mediated (91, 92).

## Importance of Epitope Mapping in AR

It has been assumed that autoimmunity starts with an immune response to a single antigen, and it subsequently extends to other proteins in the same tissue, or amino acid sequence within the same molecule (epitopes) by a process called “epitope spreading” (93, 94). Testing for epitopes for major AAbs that play a role in the autoimmunity of retinopathy is important because it could potentially distinguish normal from pathogenic antibodies. There is evidence that epitope spreading plays a role in the formation of anti-retinal T cells and in antibody responses associated with ocular immunity (95). The first indication of different epitopes in the same antigenic protein involved in autoimmune uveitis was demonstrated using experimental autoimmune uveitis (EAU), a model for autoimmune noninfectious uveitis in humans. Two proteins were found to be major antigens in humans and experimental animals: IRBP, also known as retinol binding protein-3, and arrestin, also known as S-Antigen (96–99). By the use of synthetic peptides of the human IRBP, the sequence within amino acids 1–20 was identified as a major uveitogenic epitope for the T cell response, which is widely recognized by different species (100). Two other IRBP epitopes, residues 461–480 and 651–670, are also uveitogenic (101). The study of EAU have shown that it is important to use human protein sequences in epitope mapping for human immune responses because the amino acid differences between experimental animals and human sequences may affect the identification of novel pathogenic epitopes and the determination of epitope spreading. In the EAU model, epitope spreading from the immunizing to the non-immunizing peptide was demonstrated for both IRBP 1–20 and 629–643 sequences and was consistent with the destruction of retinal tissue, increasing repertoire of antigen-specific effector T-cell with disease progression (102).

Arrestin epitopes were studied in patients with uveitis, using linear synthetic peptides, spanning the entire sequence of the protein. Two synthetic peptides, amino acid regions 286–305 and 306–325 are uveitogenic in the rat model of uveitis (103). Human patients respond to the arrestin peptides 61–80 and other arrestin peptides with high frequencies (104). These results further confirm that autoimmunity to arrestin is crucial for the pathology of uveitis (104). In the case of IRBP and arrestin proteins, the demonstration of epitope spreading is correlated with clinical disease, which implies that an evolving immune response plays an important part in progression of chronic autoimmune ocular inflammation. AAbs to arrestin and IRBP are not only important in autoimmune uveitis but they are detected in AR and CAR (105).

Recoverin was one of the first presumed autoantigen in CAR. It has been demonstrated in *in vitro* and *in vivo* studies that this protein is immunogenic and induces the production of pathogenic antibodies and T cells (59, 62, 106–108). In epitope mapping experiments, using synthetic peptides that overlap the entire recoverin sequence, two major epitopes for human AAbs have been found within the residues 38–43 (QFQSI) and 64–70 (KAYAQHV), in proximity to the calcium-binding domain EF-hand 2 (25, 107). Both mapped regions of recoverin can be accessible to the immune system because they exist on the surface of the molecule. AAb binding was found to be dependent on recoverin calcium-binding properties, which induce conformational changes in the recoverin protein, and enhance binding of AAbs to recoverin (107). The majority of antibodies to recoverin, both in human disease and in animals that are immunized with recoverin, are directed against the same major immunodominant region, the sequence 64–70 (107, 109). An immunization of animals, with the peptide 64–70, induced EAU in rats and the antibodies that were generated affected photoreceptor cell function, which in turn produced an activation of the caspase-dependent apoptotic pathways *in vitro* (25, 106, 109, 110).

Cancer-associated retinopathy patients possess recoverin-specific cytotoxic T lymphocytes (CTLs) in the peripheral blood, which can recognize aberrantly expressing recoverin in cancer cells (109, 111). In the study of recoverin-derived HLA class I—A24-binding peptides to generate antitumor-recoverin CTLs, the investigators identified three recoverin epitopes: R49 (QFQSIYAKF), R49.2 (QFQSIYAKFF), and R64 (AYAQHVFRSF) (109). In part, these CTL epitope sequences (QFQS and AYAQHV) correspond to the major binding sites for anti-recoverin AAbs in humans (107). Overall, it suggested that the capacity to induce the pathogenic effects is dependent on the region of the antigenic protein that is being recognized by the immune system.

Enolase-α is a major antigen in CAR and AR. Soon after its discovery, there was a concern about a broad association of anti-enolase-α AAbs in an autoimmunity that is not restricted to any particular disease, and is occasionally found in sera of normal individuals (36, 112). Epitope mapping of CAR and normal sera revealed three binding regions of enolase within the amino acid residues 31–38 (FRAAVPSG), 176–183 (ANFREAMR), and 421–428 (AKFAGRNF), and these epitopes were common for all AAbs tested, independent of disease status (113). However, the enolase sequence 56–63 (RYMGKGVS) is uniquely recognized by CAR sera. There are also differences in *in vitro* cytotoxic activities and cell-death-promoting activities between anti-enolase AAbs of healthy and CAR affected individuals (113). Anti-enolase-α AAbs from patients with CAR and healthy individuals did not bind to the epitope involved in the plasminogen binding of enolase-α sequence 250—256 (FFRSGKY), suggesting that these conditions are not associated with distresses of the intravascular and pericellular fibrinolytic system (114).

Anti-enolase AAbs have been associated with a number of cancer and autoimmune diseases but there is limited information about the fine recognition of this antigen. Anti-enolase AAbs of patients with endometrial adenocarcinoma, but not from healthy individuals, were found to recognize 2 regions of enolase sequence: 53–87 and 207–238 (115). The first epitope that was associated with endometriosis, sequence 53–87, overlaps with the presumed pathogenic epitope related to CAR, sequence 56–63 (115). One study showed that the common human enolase sequence 257–272 (DLDFKSPDDPSRYISP), spanning amino acids located within an external loop of the molecule, showed similarities between α-enolase and α-ERM molecule (116). The high specificity of antibodies to the NH_2_-terminal region of enolase-α in patients with Hashimoto’s encephalopathy suggests that this is the most immunogenic site and that AAbs against N-terminal enolase can serve as diagnostic biomarkers for the disease (117). The NH_2_-terminal region of α-enolase is located on the external part of the enzyme and is important for intermolecular interactions (118). The overall conclusion, based on those findings, is that in spite of recognition of enolase-α, AAbs that are found in different diseases and in normal individuals are not the same—they can bind to different parts of molecule.

The study of fine specificity of AAbs generated against CAII further confirms that AAbs against common proteins are distinctive. During the course of retinal disease from non-paraneoplastic (AR) to paraneoplastic stage (CAR), sera bind to different domains of CAII and differ from healthy control sera (33). The AR sera predominantly react with the following N-terminal epitopes: 22–26 (IAKGE) and 85–90 (DGTYRL), which corresponded to the catalytic core of the enzyme. The major epitopes for CAR AAbs are found to be reactive with the peptide 201–208 (CVTWIV) and 218–222 (SSEQVL) clustered with the α-helix, and the last sheet strand 17, the peptide 254–258 (RQIKA). It is important to note that the N-terminal epitope 85–90 is recognized by 91% of AR patients and the major epitope for CAR is the sequence 218–222 that reacts with 77% of patients. The analysis of epitope location in a 3D molecular structure of the native CAII reveals their partial or full exposure on the protein surface. Anti-CAII AAbs from normal healthy controls do not share the major determinants with CAR and AR patients. This remarkable finding showed the shift in epitope recognition from the primary AR-like epitope profile to the secondary CAR-like antibody profile in a patient who developed cancer 2 years after initial symptoms of vision loss (33). The detailed knowledge of epitopes recognized by anti-retinal AAbs that are associated with retinal paraneoplastic syndromes is valuable for development of new diagnostic assays for CAR, MAR, and AR, and for the study of mechanisms involved in pathogenesis of retinopathy.

## Pathogenicity of Anti-Retinal AAbs

**Figure 4:**
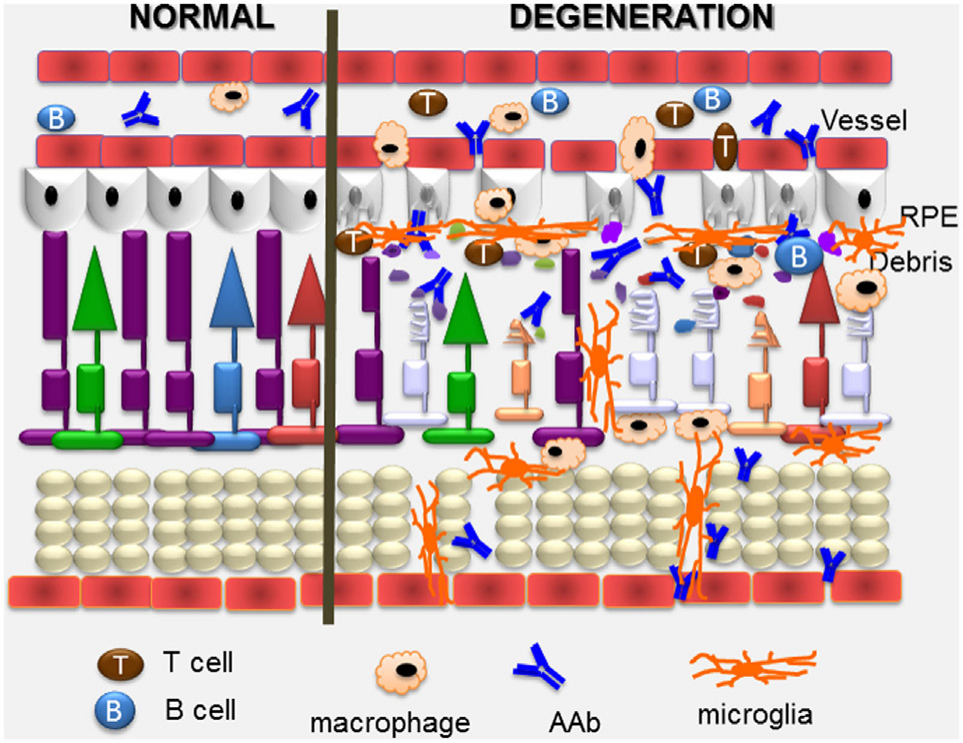
Overview of pathogenic autoimmune processes on the retina. Anti-retinal autoantibodies (AAbs) can provoke and can be a consequence of retinal degeneration. AAbs attract activated microglia/microphages that produce pro-inflammatory cytokines and chemokines, and induce apoptosis of cone and rod photoreceptor cells (showing on the right), resulting in retinal degeneration. Inflammatory cytokines and chemokines, activation of microglia and photoreceptor apoptosis can trigger secondary autoimmunity, and production of new anti-retinal AAbs. Cytotoxic T cells specific for retinal antigens are likely also involved in the pathogenic process.

Autoantibody-mediated retinal injury can be mediated by cytotoxic T cells and AAbs to detect self-antigens in the cell (109). The perceived inability of anti-retinal AAbs to get to the retina and cross cell membranes to access their target intracellular antigens diminished the understanding of their role in the pathogenicity of retinopathy (Figure 4). However, a number of studies have demonstrated that anti-retinal AAbs can indeed penetrate the cells and affect their viability and cellular function (74, 80, 108, 113, 119–123). Given the great diversity of anti-retinal AAbs, it is likely some antibodies have greater pathogenic potential than others to impact retinal pathophysiology.

It has been suggested that the AAbs reactivity with target cells depends on the microenvironment and genetic traits of individual (108, 119, 122, 124). AAbs alone might have little or no effect on healthy cells, but can be highly cytotoxic when coupled with other damaging conditions, like inflammation or cancer treatment. The first step in antibody pathogenicity is the ability to get to the antigen. As majority of antigens are intracellular, AAbs have to penetrate the tissue to get to the target cells. The fact that they can penetrate into living cells can explain their pathogenic potential, functioning through the activation of cell apoptosis after prolonged exposure of the retina to such antibodies from the circulation (125). Not only anti-recoverin IgGs but also their Fab fragments penetrate into retinal cells *in vitro* and *in vivo* by an active process and induce apoptosis through the caspase 3 pathway (108, 122, 126). One study showed that the mechanism of cellular entry by an AAb can be mediated by a membrane proxy protein for the target antigen, e.g., the cell surface molecules, calreticulin, and myosin-1 can serve as surrogate antigens for penetrating anti-double-stranded DNA antibodies (127).

In retinopathy, AAbs have different antigenic specificities and may affect different metabolic pathways, including phototransduction for recoverin, glycolysis for enolase, and pH control for CAII, they all cause an increase in the intracellular calcium (Table 1), which may be an initial step that triggers cell death (36, 128). Such a calcium-induced cytotoxicity seems to play a major role in AR and several other diseases.

**Table 1:** Effects of major CAR autoantibodies (AAbs) on retinal cell function *in vitro*.

AAb	Autoantigen	Effect on function	Mechanism
Anti-recoverin	23-kDa calcium-binding protein present in photoreceptor cells; regulates rhodopsin phosphorylation in a calcium-dependent manner in phototransduction cascade	AAbs deregulate the phototransduction, leading to retinal degeneration in CAR when access retinal cells	Inhibition of calcium-binding function
			Elevation of intracellular calcium
			Deregulation of phototransduction

Anti-α-enolase (likely anti-GAPDH, anti-aldolase, anti-PKM2)	46-kDa enzyme present in every cell; coverts 2-phosphoglycerate into phosphoenolpyruvate in glycolytic pathway leading to ATP production	AAbs deregulate the glucose metabolism, leading apoptotic death when access retinal cells	Inhibition enolase catalytic function
			Depletion of ATP
			Elevation of intracellular calcium
			Lowering intracellular pH

Anti-carbonic anhydrase II (CAII)	30-kDa enzyme present in the retina; catalyzes a reversible conversion of carbon dioxide to bicarbonate and participle in pH control and ion transport	AAbs impair the pH control and ion transport, leading to cell dysfunction and death when access cells	Inhibition of catalytic activity of CAII in a dose-dependent manner
			Decrease of intracellular pH
			Increasing intracellular calcium

Normal Abs	Unknown	None	None

It is conceivable that after cellular entry, anti-recoverin AAbs bind the antigen and block its function, which regulates rhodopsin phosphorylation in a Ca^2+^-dependent manner (129). Interestingly, the anti-recoverin antibody promotes rhodopsin phosphorylation when added to rod outer segments of photoreceptor cells *in vitro* (123). Similar effects on rhodopsin phosphorylation are observed in rat eyes treated with anti-recoverin antibody, which in effect impairs the ERG responses. Co-injection of caspase inhibitors with anti-recoverin significantly suppresses the antibody-induced enhancement of rhodopsin phosphorylation, suggesting that these antibodies dysregulate cellular function. After anti-retinal antibody entry, there is a rapid increase in the intracellular [Ca^2+^], which results in the activation of the mitochondrial apoptotic pathway followed by cell death (128, 130). Not only anti-recoverin but also anti-rhodopsin, anti-arrestin, and anti-enolase AAbs have the ability to induce the influx of intracellular calcium and cytotoxicity (128). In consequence, AAbs could influence cell activation, proliferation, and death and induce expression of pro-inflammatory cytokines (131). It was also reported that anti-recoverin antibodies were found in the aqueous humor and vitreous cavity beyond the BRB in a patient with CAR. Altogether, the experimental findings suggest that antibody-mediated retinal cell death involves multistep process, in which anti-recoverin AAbs must reach the retina from the circulation and then penetrate photoreceptor cells, block the recoverin function, increase intracellular calcium, and enhance of rhodopsin phosphorylation, which in effect activates apoptosis (108, 122, 123, 132).

In addition to anti-recoverin AAbs, anti-enolase and anti-CAII antibodies also block the function of their corresponding proteins. Most anti-enolase antibodies bind to Müller cells and ganglion cells and they act on those cells, inducing apoptotic death (74). After anti-enolase antibody cellular internalization, the antibody inactivates the enzyme (133). However, the enzymatic activity of enolase is not inhibited when the retinal cells are treated with endocytosis inhibitor cytochalasin B, which blocks antibody entry into the cells. The anti-enolase antibody blocks the catalytic function and likely inhibits glycolysis by reducing glycolytic ATP. Such changes in ATP, as an effect of antibody action, over the lifetime of a patient, may slowly damage neuronal cells and lead to retinal degeneration. In fact, in some patients with anti-enolase AAbs, visual impairment has a slow progression of central vision loss (11). In experiments using *ex vivo* eye explants and intravitreal injections of anti-enolase AAbs, the enolase antibodies are also capable of penetrating retinal tissue to target ganglion cells and inner nuclear layers and consequently induce cell-containing antigen apoptosis (62). The apoptotic nuclei detected by a DNA fragmentation assay and caspase 3-positive cells are co-localized to their targeted retinal layers—the ganglion cell layer and inner nuclear layer. Normal AAbs without specificity to retinal antigens did not induce pathogenic processes in retinal cells. A similar mechanism may occur in patients with CAR, which may lead to visual loss and blindness. Figure 4 illustrates the presumed autoimmune pathogenic process in retinal degeneration.

Anti-CAII AAbs have also the capacity to induce cellular damage by impairing CAII cellular function through inhibiting the catalytic activity of CAII in a dose-dependent manner, decreasing intracellular pH, and increasing intracellular calcium, which in effect decreases retinal cell viability and survival (134). The decrease of intracellular pH, related to the function of the enzyme in the cell could cause acidification by changes in CO_2_/H_2_CO_3_ metabolism and in proton pump activity, the Na^+^/H^+^ exchanger. Some anti-CAII AAbs cause a decrease of pH to below pH 6.8, which could lead to caspase activation in those retinal cells. The destabilized catalytic function of CAII and alterations in cytosolic pH occur very early in the process, suggesting that AAbs are the inducers of apoptosis (77).

In addition to the *in vitro* studies (above), animal investigations further confirmed that anti-recoverin antibodies have pathogenic potential. After intravitreal injection into the eye, anti-recoverin antibodies are capable of penetrating photoreceptor and bipolar cells, the normal site of recoverin expression in the retina, and then they induce apoptotic cell death as detected by an increased number of TdT-dUTP terminal nick-end labeling (TUNEL)-positive cells, fragmentation of DNA in a DNA ladder electrophoresis, and structural changes observed in electron microscopy (107). Intravitreal injection of AAbs against recoverin, together with anti-heat shock cognate protein 70 (HSP70) that co-exist in patients with CAR, cause a decrease in ERG amplitudes and apoptotic cell death in the outer nuclear layer in Lewis rat eyes (113). Internalization of anti-HSP70 AAbs into photoreceptor cells likely blocks the biologic protection of HSP70 chaperon functions to suppress protein aggregation, denaturation, and misfolding, which in effect may promote anti-recoverin antibody-mediated retinal degeneration. Synergetic autoimmune responses against recoverin and HSP70 suggest their involvement in the pathogenesis of CAR (62).

In the recent years, AAbs associated with retinopathy in melanoma patients (MAR) caught the attention of scientists. In the study of ret-transgenic mice that spontaneously develop melanoma, the investigators observed that those transgenic mice frequently developed retinopathy (28). Passive transfer of MAR sera or spleen cells also induce morphological changes in the retina and stimulates functional changes as measured by the ERG in recipient mice (28). These pathological changes reveal degeneration of photoreceptors, bipolar cells, and pigment epithelium as well as produce retinal detachment. Some of the mice with melanoma tumors also had AAbs against arrestin and transducin.

Investigations of TRPM1 AAbs have shown that incubation of living retinal neurons with TRPM1-positive MAR serum cause accumulation of IgGs in ON bipolar cells isolated from TRPM1-positive mice, but not knockout TRPM1^−/−^ mice, suggesting that the uptake of AAbs targets the intracellular antigen, which in effect reduces the ON bipolar cell response to light (58, 80). MAR AAbs reduce the b-wave amplitude of the ERG by targeting a key component of the ON bipolar cell signal transduction pathway (80). After intravitreal administration of TRMP1-positive serum into mice eyes, the ERGs are altered acutely and antibodies are detected in bipolar cells, but only in wild-type and not in TRPM1-knockout mice (79). Moreover, the bipolar cells appear apoptotic within 5 h after the injection and in 3 months, the inner nuclear layer was thinner and the amplitudes of the ERGs were more reduced. Recently published studies revealed cross-reactivity of TRPM1 with TRPM3, which is present in the RPE, suggesting that such AAbs could contribute to pathology of MAR, possibly inducing retinal and RPE detachments (56).

It has been argued that if healthy individuals and affected patients are seropositive for the same AAbs, those AAbs cannot be pathogenic. However, studies on epitope mapping for anti-recoverin, anti-enolase, and anti-CAII reveal that although different AAbs recognize the same protein, they bind to a different immunogenic domain on the molecule (33, 107, 113). Epitope mapping shows that natural antibodies may have similar antigenic specificity to CAR and AR AAbs, but they can still be distinguished based on their fine epitope recognition pattern (see above). Natural antibodies are IgM and IgG classes, are usually cross-reactive with antigens of different origin (polyspecific) and recognize non-self-antigens of pathogens as well as self-related antigens (135, 136). It is important to appreciate that natural antibodies provide various essential functions within the immune system, such as providing protection against infections and removing cellular debris (137).

## Final Remarks

A number of studies have revealed the existence of various retinal autoantigens that specifically interact with AAbs in patients with autoimmune retinopathies. Such a vast repertoire of anti-retinal AAbs observed in patients with CAR, AR, and AR is not surprising because other autoimmune diseases are associated with multiple AAbs. Anti-retinal AAbs, especially those that were made against cancer or microbial cross-reactive antigens, are likely to play a primary causative role in retinal degeneration, whereas others can contribute to disease progression and promote damage of retinal cells. It has been argued that anti-retinal AAbs are a result of retinal demise rather than a direct cause (epiphenomenon) of retinopathy. Thus, dying photoreceptors by apoptosis that is initiated by some other mechanisms (hereditary), produce substantial amounts of debris, containing high concentrations of antigens discharged from dying outer segments, and which may lead to autoimmunization and the enhanced permeability of the blood–retinal barrier (138, 139). Although not all antigens have been identified and are known only by their molecular mass, they can be used to follow the progression of retinopathy and the effects of treatment. It is important to remember that some anti-retinal AAbs can occur before the diagnosis of cancer, so they could be used in early cancer detection, e.g., anti-recoverin AAbs (25). Moreover, they can also serve as biomarkers in the context of ocular presentation and findings, despite the fact that the pathogenicity has not been conclusively proven.

## Author Contributions

The sole author is totally responsible for the design and writing of the review.

## Conflict of Interest Statement

The author declares that the research was conducted in the absence of any commercial or financial relationships that could be construed as a potential conflict of interest.

## Abbreviations

AAbs, autoantibodies; AR, autoimmune retinopathy; CAR, cancer-associated retinopathy; MAR, melanoma-associated retinopathy; RCS, Royal College of Surgeons; RPE, retinal pigment epithelium; APC, antigen-presenting cells; GCAP, guanylate cyclase-activating protein; TULP1, tubby-like protein 1; IRBP, interphotoreceptor retinoid-binding protein; HSP27, heat shock protein 27; CAII, carbonic anhydrase II; TRPM1, transient receptor potential channel protein 1; TULP1, tubby-like protein 1; ERG, electroretinogram.
